# Identification and antifungal susceptibility of *Candida* species isolated from bloodstream infections in Konya, Turkey

**DOI:** 10.1186/s12941-016-0153-1

**Published:** 2016-05-31

**Authors:** Hatice Turk Dagi, Duygu Findik, Cigdem Senkeles, Ugur Arslan

**Affiliations:** Department of Microbiology, Faculty of Medicine, Selcuk University, Alaeddin Keykubat Campus, 42131 Konya, Selcuklu Turkey

**Keywords:** *Candida*, Anti-fungal susceptibility, Microdilution method

## Abstract

**Background:**

In this study, our aim was to identify *Candida* species isolated from bloodstream infections and to determine their susceptibilities to various antifungal agents to demonstrate the local resistance profiles and to guide empirical treatment for clinicians.

**Methods:**

Two hundred *Candida* isolates (95 *Candida albicans*, 105 non-albicans *Candida* strains) were included in the study. *Candida* species were identified by conventional, biochemical and molecular methods. Antifungal susceptibility tests for amphotericin B, fluconazole, voriconazole, posaconazole, caspofungin and anidulafungin were performed with broth microdilution method according to the Clinical and Laboratory Standards Institute M27-A3 document.

**Results:**

Of the 200 *Candida* strains, the most prevalent species were *C. albicans* (47.5 %), *Candida glabrata* (18.0 %) and *Candida parapsilosis* complex (14.0 %). All *Candida* species except for three (1.5 %) *Candida kefyr* strains were susceptible to amphotericin B. Only one (2.8 %) *C. glabrata* was resistant to fluconazole (MIC ≥ 64 μg/ml), and the others (97.2 %) exhibited dose-dependent susceptibility. All species, but *C. glabrata* strains, were susceptible to fluconazole. Resistance to voriconazole, posaconazole, caspofungin and anidulafungin was not detected in any strain.

**Conclusion:**

*Candida albicans* were susceptible to all antifungal drugs. Three *C. kefyr* strains were resistant to amphotericin B. Only one *C. glabrata* was resistant to fluconazole. All the strains were susceptible to voriconazole, posaconazole, caspofungin and anidulafungin. In vitro antifungal susceptibility tests should be performed to select of appropriate and effective antifungal therapy, and monitor the development of resistance.

## Background

*Candida* species are common in nature and the flora of the human skin and mucosa and have been reported more frequently as pathogens. This is because of the risk factors such as increased use of broad-spectrum antibiotics, underlying malignant diseases, HIV/AIDS, organ transplantation, prolonged hospital stay, and exposure to invasive procedures, various opportunistic fungal infections have increased [1, 2].

*Candida* species can lead to a wide range of serious infections including blood stream infections (BSIs) and disseminated candidiasis. *Candida* species are fourth most frequent pathogens in BSIs. In spite of the advances in the diagnosis and treatment of candidiasis, the infections still have high mortality rates [3, 4].

In recent years, a gradual increase in the fungal diseases and the widespread use of empirical antifungals caused the emergence of resistant strains of fungi. Therefore, in vitro antifungal susceptibility testing requirements are increasing to select appropriate and effective antifungal therapy. The main purpose for using these tests to enable to anticipate the clinical success during the treatment of infections [5].

Antifungal susceptibility tests provide useful information to the clinicians about empirical treatments. There are some studies on the antifungal susceptibility of *Candida* species with broth microdilution method from Turkey [6–8], but there is no published research evaluated according to species-specific clinical breakpoints in the Clinical and Laboratory Standards Institute (CLSI) M27-S4 document. In this study, our aim was to identify *Candida* species isolated from BSIs and to determine the susceptibility to various antifungals including amphotericin B, fluconazole, voriconazole, posaconazole, caspofungin, and anidulafungin to demonstrate the local resistance profiles and to guide empirical treatment for clinicians.

## Methods

This study was approved by Ethical Committee of Faculty of Medicine, Selcuk University (2011, 13).

A total of 200 *Candida* isolates (*Candida albicans*: 93, *Candida glabrata*: 36, *Candida parapsilosis* complex: 28, *Candida tropicalis*: 24, *Candida kefyr*: 10, *Candida lusitaniae*: 7, and *Candida dubliniensis*: 2) were collected between 2010 and 2013 from blood cultures of hospitalized patients in various departments of the Selcuk University, Faculty of Medicine. *Candida* strains isolated from the clinical samples were identified by conventional methods (germ tube test, morphology on corn meal agar) and API ID 32C (bioMerieux, France) according to the manufacturer’s instructions. The isolates were stored at −70 °C in the Brain Heart Infusion broth (Oxoid, United Kingdom) with 20 % glycerol until they were studied.

After the stored isolates were subcultured, DNA was extracted using a commercial DNA isolation kit (Gentra Puregene Yeast/Bact. Kit, Qiagen, Valencia, CA, USA) according to the manufacturer’s recommendations. The primers (CR-f 5′-GCTACCACTTCAGAATCATCATC-3′ and CR-r 5′-GCACCTTCAGTCGTAGAGACG-3′) encoding hypha l wall protein 1 (HWP1) gene were selected to identify *C. albicans* and *C. dubliniensis* correctly. The PCR conditions were performed as described before [9]. PCR amplification products were electrophoresed on 1.3 % (wt/vol) agarose gel and visualized by staining with ethidium bromide (0.5 µg/mL) using a GelDoc imaging system (BioRad, Hercules, CA, USA). The isolates yielded a DNA fragment of 1000 bp were identified as *C. albicans*. The molecular identification of *C. parapsilosis* complex was performed by sequencing the internal transcribed spacer (ITS) region with ITS1 (5′-TCCGTAGGTGAACCTGCGG-3′) and ITS4 (5′-TCTTTTCCTCCGCTTATTGATATG-3′) primers as previously described [10]. The amplicons were purified with commercial kit (QIAquick PCR Purification Kit, Qiagen, Valencia, CA, USA) and were analyzed by the use of an ABI 3100 Genetic Analyzer (Applied Biosystems, Foster City, CA, USA). The sequences were evaluated using the Sequencer 5.3 software and compared with GenBank.

Antifungal susceptibility tests were performed by broth microdilution method as described in the CLSI M27-A3 document [11]. The following antifungals were used: amphotericin B, fluconazole, voriconazole, posaconazole, caspofungin (Sigma-Aldrich, St. Louis, MO, USA) and anidulafungin (Pfizer, New York, NY, USA). The antifungal agents and concentration ranges were between 0.015 and 16 µg/mL for amphotericin B, voriconazole and posaconazole; 0.12–64 µg/mL for fluconazole; 0.008–8 µg/mL for anidulafungin and caspofungin. The minimum inhibitory concentration (MIC) values for all agents were read following 24 h of incubation at 35 °C. The MIC values were visually determined at the lowest concentration of drug that prevents any noticeable growth for amphotericin B and a significant reduction of growth (≥50 %) for the azoles and the echinocandins compared with the drug-free growth control. Species-specific clinical breakpoints in the M27-S4 document were used for categorical evaluation [12]. Due to the lack of published breakpoints by CLSI for posaconazole, the voriconazole breakpoints were used for posaconazole. The isolates had ≤1 µg/mL MIC for AmB were accepted susceptible according to CLSI M27-S3 [13]. *C. parapsilosis* ATCC 22019 and *C. krusei* ATCC 6258 were used as quality control strains.

## Results

A total of 93 *C. albicans* and two *C. dubliniensis* isolates were identified as *C. albicans* by HWP1 gene polymorphisms. The 28 *C. parapsilosis* complex isolates were sequenced, and all of the strains were identified as *C. parapsilosis.* None of the *C. parapsilosis* complex strains was identified *Candida metapsilosis* or *Candida orthopsilosis*.

Antifungal susceptibility results and MIC values as well as categories of the isolates were presented in Table 1. All *Candida* species except for *C. kefyr* were susceptible to amphotericin B. MIC values of three *C. kefyr* strains were 2 µg/mL for AmB. AmB had the lowest MIC_90_ value (0.25 µg/mL) against *C. albicans* and *C. parapsilosis.* Only one *C. glabrata* strain was resistant to fluconazole (MIC = ≥64 μg/mL), and the others showed dose-dependent susceptibility. The other *Candida* species were susceptible to fluconazole. All strains were susceptible to voriconazole, posaconazole, caspofungin and anidulafungin. Voriconazole and posaconazole had the same MIC_90_ value (0.06 µg/mL), but posaconazole had the lower MIC_50_ value (≤0.015 µg/mL) than voriconazole (0.03 µg/mL). By having MIC_90_ values of 0.06 µg/mL, caspofungin and anidulafungin were potentially active agents against *Candida* species.

**Table 1:** In vitro susceptibilities of *Candida* species to antifungal agents determined by the broth microdilution method

Species (number) and antifungals	MIC range (µg/ml)	MIC_50_ (µg/ml)	MIC_90_ (µg/ml)	S (%)	S-DD or I (%)	R (%)
*Candida* spp. (200)						
Amphotericin B	0.06–2.0	0.12	0.5	100	–	0
Fluconazole	0.12– ≥ 64.0	0.25	1.0	–	–	–
Voriconazole	≤0.015–0.12	0.03	0.06	–	–	–
Posaconazole	≤0.015–0.12	≤0.015	0.06	–	–	–
Caspofungin	≤0.008–0.12	0.015	0.06	–	–	–
Anidulafungin	≤0.008–0.12	0.03	0.06	–	–	–
*C. albicans* (95)						
Amphotericin B	0.12–1.0	0.12	0.25	100	–	0
Fluconazole	0.12–2.0	0.25	0.5	100	0	0
Voriconazole	≤0.015–0.06	≤0.015	0.03	100	0	0
Posaconazole	≤0.015–0.12	≤0.015	0.03	100	0	0
Caspofungin	≤0.008–0.12	0.015	0.06	100	0	0
Anidulafungin	0.015–0.12	0.015	0.03	100	0	0
*C. glabrata* (36)						
Amphotericin B	0.06–1.0	0.12	0.5	100	–	0
Fluconazole	0.12– ≥ 64.0	1.0	4.0	–	97.2	2.8
Voriconazole	≤0.015–0.12	0.03	0.06	–	–	–
Posaconazole	≤0.015–0.12	0.06	0.12	–	–	–
Caspofungin	≤0.008–0.12	0.06	0.12	100	0	0
Anidulafungin	0.015–0.12	0.03	0.06	100	0	0
*C. parapsilosis* (28)						
Amphotericin B	0.06–0.5	0.12	0.25	100	–	0
Fluconazole	0.12–0.5	0.12	0.5	100	0	0
Voriconazole	≤0.015–0.03	≤0.015	0.03	100	0	0
Posaconazole	≤0.015–0.03	≤0.015	0.03	100	0	0
Caspofungin	0.015–0.12	0.015	0.06	100	0	0
Anidulafungin	0.015–0.12	0.015	0.06	100	0	0
*C. tropicalis* (24)						
Amphotericin B	0.12–0.5	0.25	0.5	100	–	0
Fluconazole	0.12–1.0	0.25	1.0	100	0	0
Voriconazole	≤0.015–0.06	0.03	0.06	100	0	0
Posaconazole	≤0.015–0.03	≤0.015	0.03	100	0	0
Caspofungin	≤0.008–0.06	0.015	0.06	100	0	0
Anidulafungin	0.015–0.06	0.03	0.06	100	0	0
*C. kefyr* (10)						
Amphotericin B	0.12–2.0	0.5	2.0	70	0	30
Fluconazole	0.12–0.5	0.25	0.25	–	–	–
Voriconazole	≤0.015–0.03	≤0.015	0.03	–	–	–
Posaconazole	≤0.015–0.03	≤0.015	0.03	–	–	–
Caspofungin	≤0.008–0.03	0.015	0.03	–	–	–
Anidulafungin	0.015–0.12	0.03	0.06	–	–	–
*C. lusitaniae* ^a^ (7)						
Amphotericin B	0.12–0.25			–	–	–
Fluconazole	0.12–1.0			–	–	–
Voriconazole	≤0.015–0.6			–	–	–
Posaconazole	≤0.015–0.6			–	–	–
Caspofungin	≤0.008–0.03			–	–	–
Anidulafungin	0.015–0.06			–	–	–

*S* susceptible; *S*-*DD* susceptible-dose dependent; *I* intermediate; *R* resistant

^a^A categorical evaluation was not performed because the number is smaller than ten

## Discussion

The surveillance programs have performed invasive *Candida* infections and investigated the distribution of species and antifungal susceptibility. In this study, we identified *Candida* species isolated from BSIs and determined their susceptibilities to amphotericin B, fluconazole, voriconazole, posaconazole, caspofungin, and anidulafungin using species-specific clinical breakpoints in the M27-S4 document.

*Candida albicans* is responsible for about 50 % of systemic infections caused by *Candida* species, making it the most common infectious *Candida* agent [14–16]. The changes in the distribution of *Candida* species have been observed and the rates of non-albicans species such as *C. glabrata*, *C. tropicalis* and *C. parapsilosis* have been increasingly reported. *C. albicans* still is the most common agent in many studies although its proportion significantly decreased from 64 to 45 % in Asia–Pacific and from 68 to 50 % in Europe recently [16, 17]. Even the distribution of some species shows variation in different regions of the same country. In a study from Italy, *C. parapsilosis* was higher in the center and in south than the north (25.7 vs. 19.9 %). In addition, *C. glabrata* was higher in the south than in any other region (7.5 vs. 15.9 %) [18]. In this study, the prevalence was as follows: C*. albicans*, (47.5 %) C*. glabrata* (18.0 %), *C. parapsilosis* (14.0 %), *C. tropicalis* (12.0 %), *C. kefyr* (5.0 %) and *C. lusitaniae* (3.5 %). Previous studies reported *C. albicans* is the most common agent of candidemia in Turkey [6, 7, 19], except one study reported that *C. parapsilosis* was the most common pathogen at the rate of 55.4 % in blood cultures in İzmir [20].

In recent years, the low rates of amphotericin B resistance have been reported in the *C. albicans*, *C. glabrata*, *C. parapsilosis*, *C. tropicalis*, *C. kefyr* and *C. krusei* isolates [7, 21, 22]. However, in a study conducted by Ruan et al. [23], the resistance was extremely high against amphotericin B in *C. krusei* strains (95 %) and in *C. glabrata* strains (53 %). In our study, all strains except for *C. kefyr* had MICs of ≤1 µg/mL for amphotericin B. Amphotericin B has had the lowest MIC_90_ value (0.25 µg/mL) against *C. albicans* and *C. parapsilosis.* In previous studies from Turkey, amphotericin B resistance rates ranged from 0 to 3.8 % [6, 7, 24], which was compatible (1.5 %) with this study.

Although, fluconazole is most frequently used an agent in the treatment of systemic yeast infections, resistance rates have been reported for *C. albicans* (5.7–5.8 %) and for *C. tropicalis* (6.2–9.8 %) [25–27]. Globally, *C. glabrata* showed the higher resistant rates (7.7–11.9 %) than other *Candida* species [16, 27–29]. In Turkey, resistance rates to fluconazole is low [6, 8], but the higher resistant rate (12.8 %) in *C. glabrata* was reported by another study [24]. In our study, one *C. glabrata* strain was resistant to fluconazole, the other strains (97 %) were dose-dependent susceptible. Other *Candida* species were susceptible to fluconazole.

Because of a high in vitro activity, voriconazole and posaconazole are more successful choices in the treatment of fluconazole-resistant *Candida* species [30]. Among *Candida* species, alike to fluconazole, *C. glabrata* had the highest MIC_90_ values for voriconazole and posaconazole [27, 29]. However in another study the highest resistance rates to voriconazole were determined in *C. tropicalis* (17.6 %), following *C. krusei* (7.1 %) and *C. albicans* (4.6 %) [25]. In our study, voriconazole and posaconazole had the same MIC_90_ value (0.06 µg/mL), but posaconazole had the lower MIC_50_ value (≤0.015 µg/mL) than voriconazole (0.03 µg/mL). The highest MIC values (0.12 µg/mL) were detected in *C. glabrata* isolates. All *Candida* species were susceptible to voriconazole and posaconazole. Moreover, so far, no voriconazole resistance has been reported from Turkey [6–8].

So far, although species-dependent resistance was undetermined, resistance to echinocandins in *Candida* species has been reported in some publications [28, 29, 31]. Resistance to caspofungin in *C. albicans* (0.2–0.5 %), in *C. parapsilosis* (1.9 %), in *C. tropicalis* (0.8 %) were low rates [27, 29]. However, the higher resistance rates to caspofungin between 5.1 and 7.9 % in *C. glabrata* have been reported [28, 29]. Insomuch that, *C. glabrata* isolates were resistant to caspofungin at rate of 100 %, but susceptible to anidulafungin [21]. In this study, caspofungin and anidulafungin were potentially active against *Candida* species with MIC_90_ values 0.06 µg/mL, and no resistant strain was detected. In Turkey, caspofungin resistance was reported in 14 *C. parapsilosis* isolates [8].

## Conclusion

*Candida albicans* were susceptible to all antifungal drugs. Three *C. kefyr* strains were resistant to amphotericin B. Only one *C. glabrata* was resistant to fluconazole. All the strains were susceptible to voriconazole, posaconazole, caspofungin and anidulafungin. According to our test results, there is no remarkable antifungal resistance in our hospital. Therefore, *Candida* isolates should be identified at the species level and MIC values should be determined even though very little resistance was detected. This study is the first investigation using species-specific clinical breakpoints in the M27-S4 document for categorical evaluation in Turkey. In vitro antifungal susceptibility tests should be performed to select of appropriate and effective antifungal therapy, and monitor the development of resistance.
